# Fast parameter inference in a biomechanical model of the left ventricle by using statistical emulation

**DOI:** 10.1111/rssc.12374

**Published:** 2019-09-20

**Authors:** Vinny Davies, Umberto Noè, Alan Lazarus, Hao Gao, Benn Macdonald, Colin Berry, Xiaoyu Luo, Dirk Husmeier

**Affiliations:** ^1^ University of Glasgow UK; ^2^ German Centre for Neurodegenerative Diseases Bonn Germany; ^3^ University of Glasgow and West of Scotland Heart and Lung Centre Clydebank UK

**Keywords:** Emulation, Gaussian processes, Holzapfel–Ogden constitutive law, Left ventricle heart model, Magnetic resonance imaging, Optimization, Simulation

## Abstract

A central problem in biomechanical studies of personalized human left ventricular modelling is estimating the material properties and biophysical parameters from *in vivo* clinical measurements in a timeframe that is suitable for use within a clinic. Understanding these properties can provide insight into heart function or dysfunction and help to inform personalized medicine. However, finding a solution to the differential equations which mathematically describe the kinematics and dynamics of the myocardium through numerical integration can be computationally expensive. To circumvent this issue, we use the concept of emulation to infer the myocardial properties of a healthy volunteer in a viable clinical timeframe by using *in vivo* magnetic resonance image data. Emulation methods avoid computationally expensive simulations from the left ventricular model by replacing the biomechanical model, which is defined in terms of explicit partial differential equations, with a surrogate model inferred from simulations generated before the arrival of a patient, vastly improving computational efficiency at the clinic. We compare and contrast two emulation strategies: emulation of the computational model outputs and emulation of the loss between the observed patient data and the computational model outputs. These strategies are tested with two interpolation methods, as well as two loss functions. The best combination of methods is found by comparing the accuracy of parameter inference on simulated data for each combination. This combination, using the output emulation method, with local Gaussian process interpolation and the Euclidean loss function, provides accurate parameter inference in both simulated and clinical data, with a reduction in the computational cost of about three orders of magnitude compared with numerical integration of the differential equations by using finite element discretization techniques.

## 1 Introduction

It is widely recognized that, when integrated with *in vivo* data from cardiac magnetic resonance imaging (MRI), computational modelling of cardiac biomechanics can provide unique insights into cardiac function in both healthy and diseased states (Wang *et al*., 2015; Chabiniok *et al*., 2016; Gao, Aderhold, Mangion, Luo, Husmeier and Berry, 2017). For example, recent mathematical studies have demonstrated that passive myocardial stiffness is much higher in diastolic heart failure patients compared with healthy subjects (Xi *et al*., 2014). Similarly, myocardial contractility could be much higher in acute myocardial infarction patients than it is in healthy volunteers (Gao, Aderhold, Mangion, Luo, Husmeier and Berry, 2017). In particular, the myocardial passive properties not only affect left ventricular (LV) diastolic filling but also influence the pumping function in heart chamber contractions (systole) through the ‘Frank–Starling’ law (Widmaier *et al*., 2016), the relationship between stroke volume and end diastolic volume.

To assess LV function comprehensively, it is necessary to determine passive myocardial stiffness. Traditionally myocardial passive properties can be determined by a series of *ex vivo* or *in vitro* experiments (Dokos *et al*., 2002). The widely used Holzapfel–Ogden (HO) constitutive law (Holzapfel and Ogden, 2009) can give a detailed description of the myocardium response in passive state, including the effects of collagen fibre structure. However, determining the material parameters of this model is challenging for clinical applications, as one cannot perform invasive experiments as in Dokos *et al*. (2002). One possibility of estimating these parameters non‐invasively is by cardiac MRI, which allows both early and end diastolic states to be measured. We can then compare, for a given patient, these measurements with the predictions from the biomechanical model, which defines the likelihood. The biophysical parameters defining the myocardial properties (as described by the HO law) can then be inferred in an approximate maximum likelihood sense by using an iterative optimization procedure, as discussed in Gao *et al*. (2015). In the context of mathematical physiology, this procedure is referred to as solving the inverse problem.

The inverse problem itself can be solved by using a variety of methods and many studies have demonstrated that it is possible to estimate constitutive material parameters by using *in vivo* measurements even with very complex constitutive relations (Guccione *et al*., 1991; Remme *et al*., 2004; Sermesant *et al*., 2006; Sun *et al*., 2009). However, because of the strong correlation between the material parameters and sparse noisy data, the formulated inverse problem is highly non‐linear (Xi *et al*., 2011; Gao *et al*., 2015). Furthermore, determining the unknown parameters in this way is very time consuming, with the process taking days or weeks to converge, even with a modern multicore workstation (Gao *et al*., 2015; Nikou *et al*., 2016). The primary reason for this is the high computational expense of simulating from the biomechanical model, which requires a numerical integration of the underlying partial differential equations with finite element discretization. This procedure must be repeated hundreds or thousands of times during the iterative optimization of the material parameters.

As a result of the high computational costs of simulating the biomechanical model, estimating myocardial properties by using a process which uses this model as a simulator is not suitable for realtime clinical diagnosis. A potential approach to overcome this problem is emulation (e.g. Kennedy and O’Hagan (2001), Conti *et al*. (2009) and Conti and O’Hagan (2010)), which has recently been explored in the closely related contexts of cardiovascular fluid dynamics (Melis *et al*., 2017), the pulmonary circulatory system (Noè *et al*., 2017) and ventricular mechanics (Achille *et al*., 2018).

Emulation methods are far more computationally efficient as most of the computation can be done in advance, making the in‐clinic diagnosis faster. With emulation approaches, we simulate a large number of samples at different parameter specifications in advance and use these simulations combined with an interpolation method to replace the computationally expensive simulator in the optimization procedure. The choice of parameter combinations from which simulations are taken can be determined effectively by using a space filling design, in this case produced by a Sobol sequence (Sobol, 1967), to spread the parameter combinations chosen in a way that aims to maximize the information about the simulator for a given number of simulations via several uniformity conditions. Optimizing this design is an active research area (see for example Overstall and Woods (2017)), which is beyond the remit of the present paper though.

The work that is presented here is designed as a proof‐of‐concept study to assess the accuracy of alternative emulation strategies for learning the material properties of a healthy volunteer's LV myocardium based on only non‐invasive, *in vivo* MRI data. For that, we use a patient‐specific model with a fixed, patient‐specific LV geometry, and focus on the statistical methodology for biophysical parameter estimation. Additionally, we use a reduced parameterization of the HO law with the biomechanical model based on the work of Gao *et al*. (2015) in MRI data. On the basis of this approach, we compare different emulation strategies, loss functions and interpolation methods.

The first of the emulation approaches that we have tested is based on emulating the outputs of the simulator (Section 3.3.1), in this case the simulated clinical data based on the biomechanical model described. Here, individual interpolators are fitted to each of the simulator outputs, using our chosen interpolation technique. We can then calculate the loss function between the predicted output of the individual models and the observed new data points from which we wish to learn the underlying myocardial properties. Minimizing this loss function via a standard optimization routine then produces estimates of the material parameters of the new subject. A variety of loss functions can be used within our emulation methods and we have compared two different functions here. The first of these is the Euclidean loss function, which assumes independence between outputs, and the second is the Mahalanobis loss function (Mahalanobis, 1936) which allows for correlations.

The second emulation approach involves emulating a loss function rather than the outputs directly (Section 3.3.2), where again we use both the Euclidean and the Mahalanobis loss functions. For new MRI data, we calculate the loss, which quantifies the discrepancy between the model predictions and the data. Statistical interpolation is then used to obtain a surrogate loss function over the biophysical parameter space, which can be minimized with standard iterative optimization routines.

In addition to testing these two emulation paradigms, we test two interpolation techniques based on Gaussian processes (GPs) (Rasmussen and Williams, 2006). The first of these is a low rank GP emulation method, which uses the complete data set for interpolation but uses a low rank approximation to scale to high dimensions (Wood, 2003). The second method uses a local GP, where the interpolation is based on the *K*‐nearest‐neighbours that are closest to the current values of the material parameters. Using a reduced number of training points from the simulations at each stage of the optimization procedure and thereby lowering the computational costs is important, as because of the cubic computational complexity in the number of training points a standard GP would not be suitable for clinical decision support in realtime.

In this work, we firstly compare different combinations of emulation methods, interpolation methods and loss functions to determine which method provides the best estimate of the material LV properties. We do this via a simulation study (Sections 5.1, 5.2, 5.3 and 5.4), using additional independent simulations from the simulator as out‐of‐sample test data. Knowledge of the true parameter values enables us to assess the accuracy of the different combinations of methods. We then test the best combination of methods on real MRI data from the healthy volunteer from whom we have taken the LV geometry (Section 5.5), to assess the accuracy of biomechanical constitutive parameter estimation in a timeframe that is suitable for clinical applications.

## 2 Left ventricle biomechanical model

The LV biomechanical model describes the diastolic filling process from early diastole to end diastole. There are many models that can be used to describe this process and these are reviewed in detail in Chabiniok *et al*. (2016). The model that is used here is similar to those used in Wang *et al*. (2013) and Gao *et al*. (2015). The biomechanical model that was initially described in Wang *et al*. (2013) can be thought of as consisting of five parts: initial discretized LV geometry, the constitutive law (the HO law), the constitutive parameters, the finite element implementation, and corresponding initial and boundary conditions. Linking this biomechanical model to patient MRI data can allow the inference of unknown material parameters describing heart mechanics, potentially leading to improved disease diagnosis and personalized treatments (Gao, Mangion, Carrick, Husmeier, Luo and Berry, 2017).

The mathematical model takes three inputs: the initial discretized LV geometry constructed from MRI images at early diastole (Section 2.1), corresponding initial and boundary conditions (Section 2.2) and constitutive parameters (Section 2.3). Based on these inputs, the mathematical model, implemented in ABAQUS (Simulia, Providence, Rhode Island, USA), simulates the diastolic filling process by using the HO law (Section 2.3) and a finite element implementation (Gao *et al*., 2015). The output of the mathematical model then gives a model of the LV state at end diastole, which can be compared with the corresponding *in vivo* magnetic resonance images. These magnetic resonance images at end diastole are used to measure circumferential strains taken at 24 locations (these are based on the American Heart Association definition as in Gao *et al*. (2015)) and the end diastolic volume. These measurements can be compared against those generated by the biomechanical model for various constitutive parameters to learn the parameters that are associated with the volunteer from whom the magnetic resonance images were taken.

Each simulation from the mathematical model without parallelization takes about 18 min on our local Linux workstation (Intel(R) Xeon(R) central processor unit (CPU), 2.9 GHz, 32 Gbytes memory), or around 4.5 min with parallelization on six CPUs. Note that 18 or 4.5 min are required for just a single parameter adaption step of an iterative optimization, or a single addition to the emulator.

### 2.1 Initial discretized left ventricular geometry

**Figure 1:**
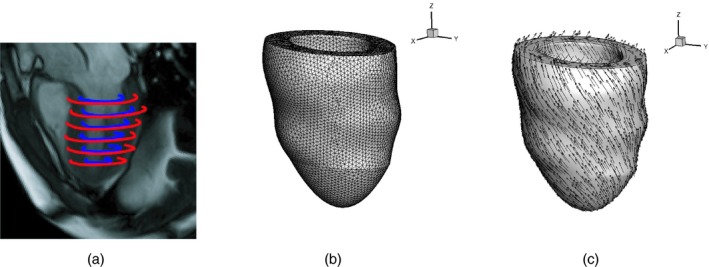
Biomechanical LV model reconstructed from *in vivo* MRI from a healthy volunteer: (a) segmented ventricular boundaries superimposed on a long axis magnetic resonance image; (b) the reconstructed LV geometry discretized with tetrahedron elements; (c) vector plot of fibre direction **f**, which rotates from endocardium to epicardium

The initial discretized LV geometry can be obtained through constructing a three‐dimensional model based on the MRI scans (Wang *et al*., 2013). The scans consist of a series of six or seven short axis cine images which cover the ventricle. (The MRI study was conducted on a Siemens MAGNETOM Avanto (Erlangen, Germany) 1.5‐T scanner with a 12‐element phased array cardiac surface coil. Cine MRI images were acquired by using the steady state precession imaging protocol. Patient consent was obtained before the scan.) For each cardiac cycle there are usually around 35 frames from end diastole to early diastole. The images of the early diastole are then used to create the initial discretized LV geometry, whereas the end diastole images will provide the final measurements of the circumferential strains and the LV volume. To create the discretized LV model, the endocardial (inner) and epicardial (outer) boundaries of the left ventricle are segmented from cine images at early diastole as done in Gao, Wang, Berry, Luo and Griffith (2014), e.g. Fig. 1(a). A three‐dimensional model of the left ventricle can then be constructed in Solidworks (Dassault Systems SolidWorks Corp., Waltham, Massachusetts, USA), e.g. Fig. 1(b). Finally, Fig. 1(c) is constructed by using a rule‐based fibre generation method (see Gao, Carrick, Berry, Griffith and Luo (2014), giving us the initial discretized LV geometry that was used in the biomechanical model. In the context of the present study, we consider this a fixed input and focus our work on developing parameter inference methods rather than a tool that can work for all possible subjects. Extensions to allow for different LV geometries is the subject of future work.

### 2.2 Initial and boundary conditions

The initial and boundary conditions, in particular LV pressure, play an important role in myocardial dynamics. Unfortunately, blood pressure within the cavity of the left ventricle can only be measured invasively, by direct catheter measurement within the LV cavity. Because of potential complications and side effects, these measurements are not available for healthy volunteers. We have therefore fixed the boundary conditions, including the pressure, at values that are considered sensible for healthy subjects, based on the work of Bouchard *et al*. (1971).

### 2.3 Constitutive law

The final part of the biomechanical model is the constitutive law for characterizing the material properties of the myocardium. In this study, we use the invariant‐based constitutive law (Holzapfel and Ogden, 2009), based on the following strain energy function:

(1) $$\begin{array}{cc} \Psi & =\frac{a}{2b}\left[\exp\left\{b\left(I_{1}-3\right)\right\}-1\right]+\sum_{i\in \{f,\;s\}}\frac{a_{i}}{2b_{i}}\left[\exp\left\{b_{i}{\left(I_{4i}-1\right)}^{2}\right\}-1\right]\\ & \;+\frac{a_{\mathrm{fs}}}{2b_{\mathrm{fs}}}\left\{\exp\left(b_{\mathrm{fs}}I_{8\mathrm{fs}}^{2}\right)-1\right\}+\frac{1}{2}K{\left(J-1\right)}^{2}, \end{array}$$

in which *a*,* b*,* a*_f_, *b*_f_, *a*_s_, *b*_s_, *a*_fs_ and *b*_fs_ are unknown material parameters, and *I*_1_,*I*_4*i*_ and *I*_8fs_ are the invariants corresponding to the matrix and fibre structure of the myocardium, which are calculated as

$$\begin{array}{cc} & I_{1}=\text{tr}\left(C\right),\\ & I_{4f}=f_{0}\cdot \left(Cf_{0}\right),\\ & I_{4s}=s_{0}\cdot \left(Cs_{0}\right),\\ & I_{8\mathrm{fs}}=f_{0}\cdot \left(Cs_{0}\right) \end{array}$$

in which **f**_0_ and **s**_0_ are the myofibre and sheet orientations, which are determined through a rule‐based approach (Wang *et al*., 2013) and are known before the simulation (initial conditions). C is the right Cauchy–Green deformation tensor, defined as $C=F^{T}F$, where F is the deformation gradient describing the motion of the myocardium and hence how its shape changes in three dimensions with time. The term $\frac{1}{2}K{\left(J-1\right)}^{2}$ accounts for the incompressibility of the material, where *K* is a constant (10^6^) and *J* is the determinant of F. The HO law forms a major part of the biomechanical model, and the eight constitutive parameters, *a*,* b*,* a*_f_, *b*_f_, *a*_s_, *b*_s_, *a*_fs_ and *b*_fs_, are unknown inputs into the model, which we wish to learn. Assessing the accuracy of parameter estimation for real data can be based on stretch–stress curves, as discussed in section 1 of the on‐line supplementary materials.

However, it has previously been found in Gao *et al*. (2015) that the eight parameters are strongly correlated, which suggests that a model reduction is advisable to ensure identifiability. They further demonstrated that myofibre stiffness, which is the parameter that is most relevant for clinical applications, can be estimated from *in vivo* data with a reduced parameterization; see section 2 of the on‐line supplementary materials. In fact, Hadjicharalambous *et al*. (2017) even estimated passive myocardial stiffness using a reduced form of the HO law with only a single unknown parameter. In the present study, similarly to Gao *et al*. (2015), we group the eight parameters of equation (1) into four, so that

(2) $$\left.\begin{array}{cc} & a=\theta_{1}\;a_{0},b=\theta_{1}\;b_{0},\\ & a_{f}=\theta_{2}\;a_{f0},a_{s}=\theta_{2}\;a_{s0},\\ & b_{f}=\theta_{3}\;b_{f0},b_{s}=\theta_{3}\;b_{s0},\\ & a_{\mathrm{fs}}=\theta_{4}\;a_{\mathrm{fs}0},b_{\mathrm{fs}}=\theta_{4}\;b_{\mathrm{fs}0}, \end{array}\right\}$$

where $\theta_{i}\in \left[0.1,5\right],\;i=1,2,3,4$, are the parameters to be inferred from *in vivo* data and *a*_0_, *b*_0_, *a*_f0_, *a*_s0_, *b*_f0_, *b*_s0_, *a*_fs0_ and *b*_fs0_ are reference values from the published literature (Gao, Aderhold, Mangion, Luo, Husmeier and Berry, 2017). (The reference values are, up to two decimal places, *a*_0_=0.22, *b*_0_=1.62, *a*_f0_=2.43, *a*_s0_=0.56, *b*_f0_=1.83, *b*_s0_=0.77, *a*_fs0_=0.39 and *b*_fs0_=1.70.) Our results obtained with this dimension reduction are consistent with the experimental results that were reported in Dokos *et al*. (2002).

## 3 Statistical methodology

This section reviews the notion of a simulator and emulator, as well as establishing the notation that is used throughout the rest of the paper. It also provides details about the emulation strategies that will be used in this paper, as well as the interpolation methods that are considered. The code for our emulation strategies, as well as the simulated data (described in Section 4), is provided at https://github.com/vinnydavies/left-ventricle-jrss-c.

### 3.1 Simulation

A *simulator* **m** is a mathematical model that relies on a computationally expensive numerical solution of the underlying system's equations. In the present study, the mathematical model is the soft tissue mechanical description of the left ventricle based on the HO strain energy function, as discussed in the previous section. The inferential process, i.e. estimating the unknown inputs or parameters ***θ***_0_ underlying the observed clinical data **y**_0_, is computationally expensive and infeasible in settings where solutions are required within a short timeframe, for instance in the context of clinical decision support. The prohibitive computational time that makes inference challenging is due to the time that is needed for a single (*forward*) *simulation* from the computational model, where by forward simulation we mean generating a (possibly multivariate) output **y**=(*y*_1_,…,*y*_*J*_)=**m**(***θ***) for a given parameter vector or input ***θ***. In the context of the present study, *J*=25, and the outputs *y*_*i*_ are the 24 circumferential strains and the LV volume at end diastole, as predicted by the mathematical model.

Given our clinical data, **y**_0_, which are the measured circumferential strains and the end‐of‐diastole LV volume obtained from MRI, we can estimate the unknown parameter vector ***θ***_0_ by finding the corresponding input to the simulator which gives rise to an output which is as close as possible to the observed clinical data **y**_0_. Although our clinical data are assumed to come from the same data‐generating process **m** for an unknown input ***θ***_0_, in practice there will be a systematic deviation due to noisy measurement and model mismatch. A standard approach to estimating the unknown input or parameter vector ***θ*** is to choose the loss function as the negative log‐likelihood:

(3) $$l\left(\theta |m,y_{0}\right)=\alpha d\left\{m\left(\theta \right),y_{0}\right\}+Z,$$

for a given metric function *d* measuring the distance between a simulation **y**=**m**(***θ***) and data **y**_0_, and some positive constants *α* and *Z*. We can then estimate the input to the model by minimizing the true loss (3):

(4) $$\hat{\theta }=\text{arg}\;\min_{\theta }l\left(\theta |m,y_{0}\right),$$

effectively giving us the maximum likelihood estimate of ***θ***. This method becomes prohibitive if a single simulation exceeds a certain amount of time, as it does with the biomechanical model that is considered in the present work. The numerical procedure based on finite element discretization requires approximately 18 min for a single simulation, or 4.5 min with parallelization on six CPUs on our computing system (a dual Intel Xeon CPU E5‐2699 v3, 2.30 GHz, 36 cores and 128 Gbytes memory). Any optimization of the true loss (3) would require the evaluation of the simulator at every iteration of the optimization routine, potentially hundreds or thousands of times, with each iteration taking between 4.5 and 18 min. This is computationally limiting if we wish to use the method for clinical decision support in realtime.

### 3.2 Emulation

An *emulator* is a statistical model that is a cheap and fast approximation to the true computational model (simulator) **m**, in this case the biomechanical model. It is used to replace the simulator to speed up both computations and inference, and it is also referred to as a *metamodel* (or *surrogate model*) as it represents a model of a model. An emulator can be built by using any interpolation technique such as regression splines, polynomial regression or GPs; see Section 3.4 for more details. Once a method has been chosen and the emulator has been fitted to the training data, we shall denote it as $\hat{m}$.

To fit a statistical model and to replace the simulator, we need training data from the simulator itself in the form of simulations $D=\left\{\left(\theta_{1},y_{1}\right),\ldots ,\left(\theta_{N},y_{N}\right)\right\}=\left\{\Theta ,Y\right\}$. In the context of the present application, the input vectors ***θ***_*i*_ are the biomechanical parameter vectors that were discussed in Section 2.3. These inputs into the simulator, **Θ**, are chosen on the basis of a space filling design, using Sobol sequences. These so‐called low discrepancy sequences are known to lead to improved convergence in the context of quasi‐Monte‐Carlo algorithms; see for example Gerber and Chopin (2015). A more efficient coverage of the input space is possible by using more advanced statistical design methods, as for example discussed in Overstall and Woods (2017), but these explorations are beyond the remit of the present work.

The outputs of the simulator, **Y**, are the resulting clinical values based on the assumed data‐generating process **m**. In the present application, the output vectors **y**_*i*_ are the vectors of 24 circumferential strains and LV volume at end diastole. Although generating large numbers of simulations is computationally expensive, this can be massively parallelized in advance and before the arrival of the patient at the clinic.

Previously, given the clinical data **y**_0_ and a simulator **m**, we could not estimate the unknown input ***θ***_0_ by using the loss function (negative log‐likelihood) given in equation (3) sufficiently fast for effective use within a clinical environment. This was due to the high simulation time that is required for each single input. Now, however, we can replace the true loss function (3) with a surrogate loss function *l* based on an emulation method; see Section 3.3 for details. Minimization of the surrogate loss (surrogate negative log‐likelihood) for any metric function *d* will be fast and suitable for realtime precision medicine, as it does not involve any simulation from the computationally expensive model.

We can use a variety of metric functions within our surrogate loss *l*. The most obvious of these is the Euclidean norm $\|\hat{m}\left(\theta \right)-y_{0}{\|}^{2}$. Under the assumption of independent and identically normally distributed errors (i.e. deviations of the clinical data from the emulator outputs) with zero mean and variance *σ* ^2^, the Euclidean loss function is equivalent to the negative log‐likelihood, up to a scaling factor and an additive constant *Z*(*σ*):

(5) $$l\left(\theta |\hat{m},y_{0}\right)=\frac{1}{2\sigma^{2}}{\left\|\hat{m}\left(\theta \right)-y_{0}\right\|}^{2}+Z\left(\sigma \right),$$

where $\hat{m}$ is the GP predictive mean, which is used to replace the expensive computational model **m**. An extension of the Euclidean loss which allows for correlations between the outputs is the Mahalanobis loss function

(6) $$l\left(\theta |\hat{m},y_{0}\right)=\frac{1}{2}{\left(\hat{m}\left(\theta \right)-y_{0}\right)}^{T}\sum^{-1}\left(\hat{m}\left(\theta \right)-y_{0}\right)+Z\left(\sum \right),$$

which is equivalent to the negative log‐likelihood of a multivariate Gaussian distribution with covariance matrix **Σ** up to a constant, *Z*(**Σ**). To minimize the computational costs at the clinic, the covariance matrix is precomputed from the training data, **Σ**=cov(**Y**), and then kept fixed. Its main purpose is to allow for the spatial correlations between the 24 circumferential strains at different locations on the left ventricle.

### 3.3 Emulation frameworks

#### 3.3.1 Output emulation

Emulating the outputs of the simulator, the LV model, involves fitting multiple individual models, one for each of the *J* outputs of the simulator **m**. These outputs, *y*_*j*_, *j*=1,…,*J*, are fitted using the inputs of the simulator, **Θ**, with an appropriate interpolation method; see Sections 3.4.1 and 3.4.2. Given the multiple independent models $\hat{m}=\left({\hat{m}}_{1},\ldots ,{\hat{m}}_{\;J}\right)$, estimates of the parameter vector ${\hat{\theta }}_{0}$ can be found for any new set of outputs **y**_0_ by minimizing the difference between **y**_0_ and $\hat{m}\left(\theta \right)$ with a loss function:

(7) $${\hat{\theta }}_{0}=\text{arg}\;\min_{\theta }l\left(\theta \;|\;\hat{m},y_{0}\right).$$

The loss function *l* in equation (7) can take a variety of forms, including the Euclidean and the Mahalanobis loss functions given in equations (5) and (6). An algorithmic description of the output emulation method is given in algorithm 1 in Table 1.

**Table 1:** Algorithm 1: inference using an emulator of the outputs

*Step 1*: simulate from the model **m**(***θ***_1_),…,**m**(***θ***_*N*_) at space filling inputs ***θ***_1_,…,***θ***_*N*_
*Step 2*: fit *J* independent real‐valued emulators $\hat{m}=\left({\hat{m}}_{1},\ldots ,{\hat{m}}_{\;J}\right)$, one for each of the *j*=1,…,*J* outputs of the simulator
*Step 3*: given data **y**_0_ and the emulator $\hat{m}$, construct the surrogate‐based loss function $l(\theta \;|\;\hat{m},y_{0})$
*Step 4*: minimize the surrogate‐based loss function to give the estimates ${\hat{\theta }}_{0}$

The advantage of emulating the outputs is that the statistical models can be fitted in advance, before the data have been collected from the clinic, meaning that, when a patient comes into the clinic, an estimation of the biomechanical parameter vector ${\hat{\theta }}_{0}$ can be carried out relatively quickly. The disadvantage is that multiple potentially correlated model outputs must be fitted, leading to higher computational costs at training time than emulating the loss function directly.

#### 3.3.2 Loss emulation

An alternative strategy is *loss emulation*. This entails direct emulation of the losses $l_{n}=l\left(\theta_{n}|m,y_{0}\right)$ rather than the simulator outputs **y**_*n*_=**m**(***θ***_*n*_), for *n*=1,…,*N*. To follow this approach we fit a single real‐valued emulator to training data:

(8) $$D_{l}=\left\{\left(\theta_{n},l_{n}\right):n=1,\ldots ,N\right\},$$

where $l_{n}=l\left(\theta_{n}|m,y_{0}\right)$ is the loss function, for a given metric *d*, evaluated at the *n*th design point from the corresponding simulation output, **y**_*n*_=**m**(***θ***_*n*_). The metric *d* should be chosen according to the problem, and it can capture the correlation between the model outputs. Now it is possible to fit a single real‐valued emulator $\hat{l}\left(\theta |m,y_{0}\right)$ of $l(\theta |m,y_{0})$ based on the training data $D_{l}$, using a single statistical model instead of a vector of model outputs. Estimation of the parameters can now be done cheaply by minimizing the emulated loss function:

(9) $${\hat{\theta }}_{0}=\text{arg}\;\min_{\theta }E\left\{\hat{l}\left(\theta |m,y_{0}\right)\right\},$$

where E denotes the conditional expectation predicted by the interpolation method, in our case the conditional mean of a GP. An algorithmic description of the loss emulation method is given in algorithm 2 in Table 2. For further illustration, an additional example, on the Lotka–Volterra system, can be found in Noè (2019).

**Table 2:** Algorithm 2: inference using an emulator of the losses

*Step 1*: simulate from the model **m**(***θ***_1_),…,**m**(***θ***_*N*_) at space filling inputs ***θ***_1_,…,***θ***_*N*_
*Step 2*: calculate the set of loss functions $l(\theta_{n}\;|\;m,y_{0})$, for *n*=1, …,*N*, between each individual simulation and the observed data **y**_0_
*Step 3*: emulate the losses by using a single real‐valued model $\hat{l}\left(\theta \;|\;m,y_{0}\right)$
*Step 4*: estimate ${\hat{\theta }}_{0}$ by minimizing the mean of the loss emulator $E\{\hat{l}\left(\theta \;|\;m,y_{0}\right)\}$

The advantage of loss emulation over output emulation is a reduction of the training complexity, as a multi‐dimensional vector is replaced by a scalar as the target function. The disadvantage is that, as opposed to output emulation, the emulator can only be trained *after* the patient has come into the clinic and the training data have become available. This implies that, on production of the training data, the emulator must be trained and the resulting emulated loss function must be optimized, leading to higher computational costs at the time that a clinical decision must be made. However, these computational costs are still low compared with running the simulator.

Loss emulation is closely related to Bayesian optimization, reviewed for example in Shahriari *et al*. (2016) and Noè (2019), which is a strategy to include further query points iteratively by trading off exploration *versus* exploitation via some heuristic or information theoretic criterion. However, every additional query point requires a computationally expensive simulation from the mathematical model, which prevents fast clinical decision making in realtime and renders Bayesian optimization infeasible for the purposes of our study.

### 3.4 Interpolation methods

We have considered several interpolation methods, based on GPs. GPs have been widely used in the context of emulation; see for example Kennedy and O’Hagan (2001), Conti *et al*. (2009) and Conti and O’Hagan (2010). For a comprehensive introduction to GPs, the reader is referred to Rasmussen and Williams (2006). Each of the interpolation methods can be used with both of the emulation paradigms that were described in Section 3.3.

#### 3.4.1 Local Gaussian process

When the sample size *N* is large, it is not feasible to use exact GP regression on the full data set, because of the *O*(*N* ^3^) computational complexity of the *N*×*N* training covariance matrix inversion. A possible approach is to use sparse GPs as in Titsias (2009), which considers a fixed number of *m* inducing variables **u**=(*u*_1_,…,*u*_*m*_), with *m*≪*N*, corresponding to inputs **Z**=(**z**_1_,…,**z**_*m*_)^T^. The locations of the inducing points and the kernel hyperparameters are chosen with variational inference, i.e. by maximizing a lower bound on the log‐marginal‐likelihood, which can be derived by applying Jensen's inequality. The computational costs of this approach are *O*(*Nm* ^2^). Initially we tried sparse GPs with 100, 500 and 1000 inducing points but, using the code accompanying the paper by Titsias (2009), the prediction time was between 0.5 and 0.6 s for 100 inducing points, around 1 s for 500 and of the order of a few seconds for 1000 inducing points (dual Intel Xeon CPU E5‐2699 v3, 2.30 GHz, 36 cores and 128 Gbytes memory). This means that minimization of the surrogate‐based loss would still be slow as approximately 1 s is required for a single evaluation. The optimization time would exceed 2.5 h for 500 inducing points when using 10000 function evaluations. With the cost of variational sparse GP models with larger numbers of inducing points being so large, we can use only about 100 inducing points to keep to our goal of realtime in‐clinic decision making. However, using such few inducing points was found to lead to around a quarter of the outputs of the biomechanical model being poorly predicted.

With the performance of the variational sparse GPs being poor when the number of inducing points is selected to give a clinically relevant decision time, we instead use a local GP approach based on the *K* nearest neighbours instead (Gramacy and Apley, 2015). This method uses the standard GP prediction formulae that were described in Rasmussen and Williams (2006), but subsetting the training data. Whenever we require a prediction at a given input, we find the training inputs representing the *K* nearest neighbours in input domain, which will form the local set of training inputs, and the corresponding outputs will represent the local training outputs. Note that, every time that we ask for a prediction at a different input, the training sets need to be recomputed and the GP needs to be trained again. However, because of the small number of neighbours *K*≪1000 that were usually selected, this method is computationally fast and accurate; see Gramacy and Apley (2015) for a discussion.

Gramacy and Apley (2015) further discussed adding a fixed number of distant points to help in the estimation of the length scale parameters, but this comes with extra computational costs required by the iterative choice of which point to add to the set of neighbours. Given the time limitations that are required by our goal (realtime clinical decision support systems) we do not pursue this approach. Furthermore, this is mostly relevant when the interest lies in building predictive models that can make good predictions when the training data are distant from each other. Since we are working on a compact set which is densely covered by the Sobol sequence, this is less relevant. For generic training data $D=\left\{\left(\theta_{1},y_{1}\right),\ldots ,\left(\theta_{N},y_{N}\right)\right\}=\left\{\Theta ,y\right\}$, we give an algorithmic description in algorithm 3 in Table 3.

**Table 3:** Algorithm 3: predicting from a local GP at θ_*_

*Step 1*: find the indices $N(\theta_{*})$ of the points in **Θ** having the *K* smallest Euclidean distances from θ_*_
*Step 2*: training inputs, $\Theta_{K}\left(\theta_{*}\right)=\left\{\theta_{1}^{\prime },\ldots ,\theta_{\;K}^{\prime }\right\}=\left\{\theta_{i}:i\in N\left(\theta_{*}\right)\right\}$
*Step 3*: training outputs, $y_{K}\left(\theta_{*}\right)=\left\{y_{1}^{\prime },\ldots ,y_{\;K}^{\prime }\right\}=\left\{y_{i}:i\in N\left(\theta_{*}\right)\right\}$
*Step 4*: train a GP by using the data $D_{K}\left(\theta_{*}\right)=\left\{\Theta_{K}\left(\theta_{*}\right),y_{K}\left(\theta_{*}\right)\right\}$
*Step 5*: predictive mean, $\hat{m}\left(\theta_{*}\right)=\mu \left(\theta_{*}\right)+k{\left(\theta_{*}\right)}^{T}{\left(K+\sigma^{2}I\right)}^{-1}\left(y_{K}\left(\theta_{*}\right)-\mu \right)$
*Step 6*: predictive variance, *s* ^2^(θ_*_)=*k*(θ_*_,θ_*_)−**k**(θ_*_)^T^(**K**+*σ* ^2^ **I**)^−1^ **k**(θ_*_)

In algorithm 3, the *K*×*K* training covariance matrix is $K={\left[k\left(\theta_{i}^{\prime },\theta_{\;\;j}^{\prime }\right)\right]}_{i,\;j=1}^{K}$, the *K*×1 vector of covariances between the training points and the test point is $k\left(\theta_{*}\right)=\left(k\left(\theta_{1}^{\prime },\theta_{*}\right),\ldots ,k\left(\theta_{\;K}^{\prime },\theta_{*}\right)\right)$ and $\mu =(\mu \left(\theta_{1}^{\prime }\right),\ldots ,\mu \left(\theta_{\;K}^{\prime }\right))$ is the *K*×1 prior mean vector. We consider a constant mean function *μ*(***θ***)=*c*. For the kernel *k*(·,·) we choose the automatic relevance determination squared exponential kernel (see for example Rasmussen and Williams (2006)), as widely used in the emulation of computer codes literature; see for example Fang *et al*. (2006) and Santner *et al*. (2003). The kernel hyperparameters are the output scale (determining the function variance) and the input length scales: one length scale for each dimension. These hyperparameters are estimated by maximizing the log‐marginal‐likelihood by using the quasi‐Newton method. The standard deviation of the additive Gaussian noise *σ* is initialized at a small value, *σ*=10^−2^, to reflect the fact that the mathematical model of the left ventricle is deterministic. (Even for deterministic models, a small non‐zero value for *σ* is usually assumed, to avoid numerical instabilities of the covariance matrix inversion.)

The CPU time that was required to obtain a prediction from the local GP is approximately 0.18 s (dual Intel Xeon CPU E5‐2699 v3, 2.30 GHz, 36 cores and 128 Gbytes memory) by using the *K*=100 nearest neighbours of a given point. The number of neighbours *K* needs to be selected on the basis of the computational time that is allowed to reach a decision in a viable timeframe, but keeping in mind that *K* also controls the accuracy of the emulation. In our experiments we found that *K*=100 was sufficiently fast for the method to be applicable in the clinic while leading to accurate predictions at the test inputs, as discussed below in Section 5.

For this method, the surrogate‐based loss and the emulated loss were optimized by using the global search constrained optimization algorithm by Ugray *et al*. (2007), over the bounded domain [0.1,5]^4^, which is implemented in MATLAB's Global Optimization toolbox. (Available from https://uk.mathworks.com/products/globaloptimization.html: we use the default choice of 2000 trial points and 400 stage 1 points. Consider running a local solver from a given starting point ***θ***_0_, ending up at the point of local minimum $\hat{\theta }$. The *basin of attraction* corresponding to that minimum is defined as the sphere centred at $\hat{\theta }$ and having radius equal to $\left\|\theta_{0}-\hat{\theta }\right\|$. All starting points falling inside the sphere are assumed to lead to the same local minimum $\hat{\theta }$; hence no local solver is run and they are discarded. In simple words, stage 1 of the global search algorithm scatters initial points in the domain and scores them from best to worst by evaluating the function value and constraints. Then an interior point local solver (Byrd *et al*., 2000) is run from each trial point, starting from the point that was scored best (lowest function value), and excluding points that fall into the basins of attraction of previously found minima. When all the stage 1 points have been analysed, stage 2 generates more random points and the same procedure is run a second time.)

#### 3.4.2 Low rank Gaussian processes

Along with local GPs based on the *K* nearest neighbours, described in Section 3.4.1, we report results for another type of statistical approximation: low rank GPs, as described in section 5.8.2 of Wood (2017), whose main ideas are summarized here for generic training data $D=\left\{\left(\theta_{1},y_{1}\right),\ldots ,\left(\theta_{n},y_{n}\right)\right\}=\left\{\Theta ,y\right\}$.

Let **C**=**K**+*σ* ^2^ **I** be the *n*×*n* covariance matrix of **y** and consider its eigendecomposition **C**=**UDU** ^T^ with eigenvalues $\left|D_{i,\;i}\left|⩾\right|D_{i+1,\;i+1}\right|$. Denote by **U**_*k*_ the submatrix consisting of the first *k* eigenvectors of **U**, corresponding to the top *k* eigenvalues in **D**. Similarly, **D**_*k*_ is the diagonal matrix containing all eigenvalues that are greater than or equal to *D*_*k*,*k*_. Wood (2017) considered replacing **C** with the rank *k* approximation $U_{k}D_{k}U_{k}^{T}$ obtained from the eigendecomposition. Now, the main issue is how to find **U**_*k*_ and **D**_*k*_ sufficiently efficiently. A full eigendecomposition of **C** requires *O*(*N* ^3^) operations, which somewhat limits the applicability of the rank reduction approach. A solution is to use the Lanczos iteration method to find **U**_*k*_ and **D**_*k*_ at the substantially lower cost of *O*(*N* ^2^ *k*) operations; see section B.11 in Wood (2017). Briefly, the algorithm is an adaptation of power methods to obtain the truncated rank *k* eigendecomposition of an *N*×*N* symmetric matrix in *O*(*N* ^2^ *k*) operations. However, for large *N*, even *O*(*N* ^2^ *k*) becomes prohibitive. In this scenario the training data are randomly subsampled by keeping *n*_*r*_ inputs and an eigendecomposition is obtained for this random selection with $O(n_{r}^{2}k)$ computational cost.

**Figure 2:**
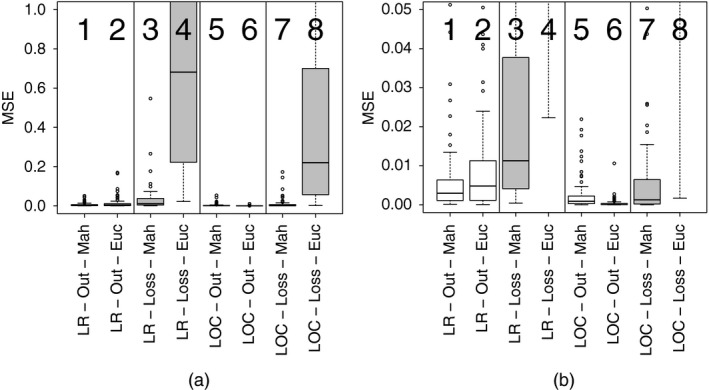
Boxplots of the MSE distribution in the prediction of all the model parameters (the methods from left to right on each plot are as follows: low rank GP (LR) output emulation (Out) with Mahalanobis loss function (Mah) and Euclidean loss function (Euc), LR–GP loss emulation (Loss) with Mahalanobis loss function and Euclidean loss function, local GP (LOC) output emulation with Mahalanobis loss function and Euclidean loss function, and LOC loss emulation with Mahalanobis loss function and Euclidean loss function; the outliers are due to non‐convergence of the optimization algorithm and the strong correlation between the parameters of the HO law): (a) boxplots of the MSE in parameter space for all the eight methods; (b) the same boxplots but with a reduced scale on the *y*‐axis

**Figure 3:**
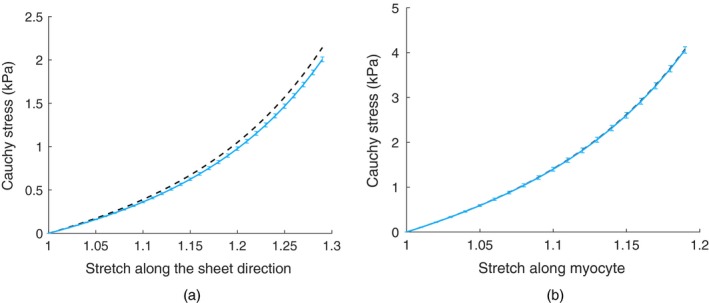
Plots of the Cauchy stress against the stretch along (a) the sheet direction and (b) the myocyte direction: 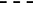, literature curves taken from the gold standard method in Gao, Aderhold, Mangion, Luo, Husmeier and Berry (2017); 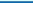, estimates of the curves from the best emulation method, the emulation of the outputs method combined with the local GP interpolation method and the Euclidean loss function; 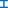, 95% confidence intervals, approximated by using the sampling method described in Section 5.5

We used the implementation that was found in the R package mgcv by Wood (2017), with the following settings: *n*_*r*_=2000 (the package default), *k*=2000 for output emulation, and *k*=1000 for loss emulation. The kernel that was used was an isotropic Matérn 3/2 kernel, with length scale set to the default of Kammann and Wand (2003): $\lambda =\max_{\mathrm{ij}}\left\|\theta_{i}-\theta_{\;j}\right\|$. The remaining model hyperparameters are estimated by maximizing the log‐marginal‐likelihood. The final model used an interaction term between each of the four model parameters, as well as a second interactive term between the inverses of the model parameters:

(10) $${\tilde{y}}_{\;j}∼\beta_{j}1+f\left(\theta \right)+f\left(\tau \right)+\varepsilon \;\text{for}\;j=1,\ldots ,J$$

where ***τ***=1/***θ***,* f*(***θ***)∼GP_LR_{*μ*(***θ***),*K*(***θ***,***θ*** ^′^)}, *f*(***τ***)∼GP_LR_{*μ*(***τ***),*K*(***τ***,***τ*** ^′^)} and GP_LR_(·) denotes a low rank GP. The model specification with the two interaction terms was found to reduce the variation in the predictive accuracy as the volume increases and the strains decrease. This can be seen in the predictions of the test and training data in Figs 2 and 3 of the on‐line supplementary materials.

Minimization of the surrogate‐based loss $l(\cdot \;|\;\hat{m},y_{0})$ and the emulated loss $\hat{l}\left(\cdot \;|\;m,y_{0}\right)$ was performed by the conjugate gradient method implemented in the R function optim (Nash, 1990), with the maximum number of iterations set to 100. To avoid being trapped in local minima, 50 different starting points from a Sobol sequence were used. The best minimum found, subject to not violating the [0.1,5]^4^ hyperinterval constraint, was kept as the estimate, discarding the remaining 49 optima.

#### 3.4.3 Multivariate output Gaussian processes

The previous two subsections have focused on single‐output GPs, while potentially correcting for the correlation structure of the outputs via a modified objective function, using the Mahalanobis distance that is defined in equation (6). We can model the correlation structure between the outputs directly via

(11) $$\text{cov}\left\{y\left(\theta_{i}\right),y\left(\theta_{\;j}\right)\right\}=K\left(\theta_{i},\theta_{\;j}\right)A$$

where *K*(***θ***_*i*_,***θ***_*j*_) is the covariance between *y*_*k*_(***θ***_*i*_) and *y*_*k*_(***θ***_*j*_) for any output *k*, and **A** is a matrix of the covariances between the outputs, i.e. the circumferential strains and the LV volume. Various approaches have been proposed in the literature. The approach that was taken in Conti and O’Hagan (2010) and Conti *et al*. (2009) is to place a non‐informative prior on **A** and to integrate **A** out in the likelihood. This leads to a closed form solution in terms of a matrix–normal distribution; see Conti and O’Hagan (2010) and Conti *et al*. (2009) for explicit expressions. However, we found that in combination with algorithm 3—to deal with the *O*(*N* ^3^) computational complexity—the computational costs of running the emulator were of the order of hours, rather than minutes, which renders this approach not viable for clinical decision support in realtime.

An alternative approach is to model the correlation structure of the outputs explicitly via

(12) $$\text{cov}\left\{y_{k}\left(\theta_{i}\right),y_{l}\left(\theta_{\;j}\right)\right\}=K\left(\theta_{i},\theta_{\;j}\right)A\left(u_{k},u_{l}\right)$$

taking into account covariates **u**_*k*_ and **u**_*l*_ associated with the *k*th and *l*th outputs, *y*_*k*_ and *y*_*l*_ respectively. Roberts *et al*. (2013) pursued this approach in the context of time series analysis, where **u**_*k*_ and **u**_*l*_ are scalar variables indicating different time points. In our application, **u**_*k*_ and **u**_*l*_ are vectors indicating the locations on the surface of the left ventricle associated with the circumferential strains. Because of the highly non‐Euclidean geometry of this space, the choice of kernel is not obvious. A naive approach that we tried is to project the locations onto a linear space defined by the first principal component (Huang, 2016). The results were not encouraging, because of the information loss that was incurred by the map. Future work could try projections onto non‐linear maps, like Hilbert curves (Hilbert, 1891; Hamilton and Rau‐Chaplin, 2007), generative topographic maps (Bishop *et al*., 1998) or self‐organizing maps (Kohonen, 1982).

A further alternative is the method of Alvarez and Lawrence (2009, 2011), who have proposed sparse convolved GPs for multioutput regression. Their method assumes that there is an underlying process which governs all of the outcomes of the model and treats it as a latent process. Modelling this latent process as a GP leads to a GP prior over the outputs, inducing cross‐covariance between the outputs and effectively introducing correlations between them. We can use the interpolation method of Alvarez and Lawrence (2009, 2011) within either of the emulation frameworks that were introduced in Section 3.3. There are, however, problems with doing this: training a convolved GP with *N* training points requires the inversion of a *DN*×*DN* matrix (where *D*=25 is the number of outputs) which is currently infeasible with all of the training data (*N*=10000), even when choosing the number of inducing points by using the method that was proposed in Alvarez and Lawrence (2009, 2011). Instead we can choose a strategy that is similar to that proposed in Section 3.4.1. This again, however, proves to be computationally expensive as fitting a single local emulator requires more than 15 min (Intel Xeon CPU E5‐606, 2.13 GHz), without consideration of the computational costs of the subsequent optmization of the LV model parameters. When this is included within either of the emulation methods (algorithms 1 and 2), the time becomes too large for a clinical decision support system, as it is infeasible to make a prediction within a clinically relevant timeframe.

Since the focus of our study is to develop an emulation framework for a clinical decision support system that can work in realtime, we have restricted our analysis to the univariate methods that were described in Sections 3.4.1 and 3.4.2.

## 4 Data and simulations

For training the emulator, we used 10000 parameter vectors generated from a Sobol sequence (Sobol, 1967) in a compact four‐dimensional parameter space, with $\theta_{1},\ldots ,\theta_{4}\in {\left[0.1,5\right]}^{4}$, where the parameter bounds reflect prior knowledge that was available from Gao *et al*. (2015). The four‐dimensional parameter vectors are then transformed to the original eight‐dimensional parameter space using transformation (2). The eight‐dimensional parameter vectors are then inserted into the HO strain energy function (1). Following the finite element discretization method that was described in Wang *et al*. (2013), the soft tissue mechanical equations are numerically solved to produce a 25‐dimensional output vector associated with each parameter vector; these are 24 circumferential strains and the LV volume at end diastole. The Sobol sequence is extended to generate an independent test set of an additional 100 parameter vectors, for which the same procedure is followed to associate them with output vectors of circumferential strains and LV volume. As a real data set, we used 24 circumferential strains and the LV volume at end diastole obtained from the cardiac MRI images of a healthy volunteer, following the procedure that was described in Gao *et al*. (2015).

## 5 Results

To summarize, we have introduced two emulation frameworks which can be used to infer the parameters of the LV biomechanical model; see Sections 3.3.1 and 3.3.2. We have applied these methods with two loss functions, the Mahalanobis loss function and the Euclidean loss function, and two interpolation methods, low rank GPs and local GPs; see Sections 3.4.1 and 3.4.2. Testing each combination of these methods means that there is a total of eight alternative procedures.

We have applied and assessed the proposed methods in a two‐pronged approach. Firstly, in Sections 5.1, 5.2, 5.3 and 5.4, we have tested the eight combinations of methods on synthetic data, where the true parameter values of the underlying biomechanical model are known; see the previous section for details on how the training and test data were generated. We compare the methods by using the mean‐square error (MSE). The distribution of 100 MSEs is given in Fig. 2 and summarized with the median and the first and third quartiles in Table 4, representing three of Tukey's five‐number summary.

**Table 4:** Median (first quartile, third quartile) of the MSE (in parameter space) in the prediction of all the model parameters†

*Interpolation*	*Emulation*	*Results for Euclidean*	*Results for Mahalanobis*
*method*	*target*	*loss function*	*loss function*
Low Rank GP	Output	0.0048 (0.0012,0.0107)	0.0030 (0.0011,0.0062)
Low Rank GP	Loss	0.6814 (0.2222,1.5234)	0.0113 (0.0041,0.0377)
Local GP	Output	*0.0001 (0.0000,0.0003)*	0.0009 (0.0003,0.0022)
Local GP	Loss	0.2201 (0.0588,0.6777)	0.0013 (0.0002,0.0063)

†The interpolation methods considered are low rank GPs and local GPs; the target of the emulation is either the model output or the loss, and two loss functions are compared, Euclidean and Mahalanobis. The method with the best predictive performance, the output emulation method with local GP interpolation and the Euclidean loss function, is given in italics.

Finally, we have applied the method with the best performance in Section 5.5 to clinical data generated from a healthy volunteer's cardiac MRI scan, where we can compare our performance against the gold standard results of Gao *et al*. (2015).

### 5.1 Comparison of interpolation methods

Looking at the two interpolation methods, the local GP method (boxplots 5–8 in Fig. 2) outperforms the low rank GP method (boxplots 1–4 in Fig. 2). The reason for the difference in performance between the two methods is the size of the noise variance that is estimated. With the low rank GP method, a larger noise variance is estimated as the interpolation must fit to the entire data set. The larger variance of the errors is in mismatch with the deterministic nature of the process that we aim to model and is the consequence of the loss of modelling flexibility resulting from the low rank approximation and a potential violation of the stationarity assumption that is intrinsic to our choice of kernel.

Conversely, with the local GP method, a much smaller error variance is estimated, which more closely matches the deterministic data generation method. This is a result of there being only a small number of points that the interpolant must fit. These points are local, giving more detail of the local surface than the low rank GP method, which uses all the points but takes a rank *k* eigendecomposition of the kernel matrix. The local GP method also provides a natural way to accommodate non‐stationary processes.

### 5.2 Comparison of emulation frameworks

Out of the two emulation frameworks, the output emulation method (boxplots 1, 2, 5 and 6 in Fig. 2) gives the most accurate parameter estimates, outperforming the loss emulation method (boxplots 3, 4, 7 and 8 in Fig. 2) for all interpolation methods and loss functions. The output emulation method provides accurate estimates for all the combinations of interpolation methods and loss functions, whereas the loss emulation method provides poor estimates in some cases. The improved parameter estimation of the output emulation method is a result of using multiple separate emulators. These multiple emulators better model the complex non‐linear relationships between the parameters and the outputs than is possible with the single emulator that is used with the loss emulation method. In the loss emulation method, the differences between the patient data and the simulations are summarized in one loss function, which entails a larger loss of information.

### 5.3 Comparison of loss functions

In terms of the accuracy of the parameter inference, the Euclidean loss and Mahalanobis loss perform differently in different emulation methods. For the loss emulation method the Mahalanobis loss function (boxplots 3 and 7 in Fig. 2) clearly outperforms the Euclidean loss function (boxplots 4 and 8 in Fig. 2) in all cases. The reason for the difference is that the loss function summarizes how similar the patient data are to the simulations and this is done more realistically by the Mahalanobis loss function in this case. This is because there are spatial correlations between the outputs due to measuring the circumferential strains at different neighbouring locations on the left ventricle. The Mahalanobis loss function accounts for this through including a correlation estimate, whereas the Euclidean loss function does not.

In comparison with the loss emulation method, for the output emulation method it is less clear which loss function gives the best results. The Mahalanobis loss function is marginally better for the low rank GP method (boxplot 1 is better than boxplot 2 in Fig. 2), whereas the Euclidean loss function gives the best performance for the local GP method (boxplot 6 is better than boxplot 5 in Fig. 2). The reason why the Euclidean loss function performs best for the local GP method is presumably because of potential inaccuracies in the covariance matrix that is used for the Mahalanobis loss function. The covariance matrix is a global measure based on the whole data set and may not accurately represent the true correlations between the local points because of limited numerical precision. (Using a local covariance matrix was also tested, but limited accuracy and numerical stability of the covariance matrix due to using only a small number of local points meant that the performance did not improve over the global covariance matrix.) This is potentially aggravated by a lack of numerical stability when inverting the covariance matrix.

### 5.4 Overall best method in simulation study

In conclusion, the results of our simulation study show the following results.

- The local GP method outperforms the low rank GP method and is the better of the two interpolation methods.
- The best emulation method is the output emulation method and this outperforms the loss emulation method in all the combinations of interpolation method and loss function tested.
- The Mahalanobis loss function gives the best performance for the loss emulation method.
- For the output emulation method, the Mahalanobis method is marginally better for the low rank GP method, but for the local GP method the Euclidean loss function gives the best parameter estimates.
- Overall, the simulation study results show that the best performing combination of methods is the output emulation method, using the local GP as the interpolation method and the Euclidean loss function (boxplot 6 in Fig. 2).

This combination of methods will be used on the cardiac MRI data of the healthy volunteer in Section 5.5.

### 5.5 Application to cardiac magnetic resonance imaging data

Fig. 2 and Table 4 show that the method which gives the most accurate parameter prediction is the emulation of the outputs method combined with the local GP interpolation and the Euclidean loss function. We have applied this strategy to estimate the material parameters for the heart model of a healthy volunteer described in Section 2, using the set of 24 circumferential strains and the LV cavity volume extracted from cardiac MRI images, as described in Section 4. The true model parameters are not known in this case, so as opposed to the simulation study we do not have a proper ‘gold standard’ for evaluation. We therefore use the following alternative procedure. We first estimate the constitutive parameters with the method of Gao *et al*. (2015) and Gao, Aderhold, Mangion, Luo, Husmeier and Berry (2017), i.e. with the method using the computationally expensive simulator. From these parameters, we calculate the stretch–stress relationships along the directions of the sheets and the myocytes, following the procedure that was described in Holzapfel and Ogden (2009). We use these graphs as a surrogate gold standard, which we compare with the corresponding graphs obtained from the parameters that were obtained with our emulation approach.

Fig. 3 shows, as broken curves, the estimate of the stretch–stress relationship for the healthy volunteer by using the gold standard method of Gao *et al*. (2015) and Gao, Aderhold, Mangion, Luo, Husmeier and Berry (2017). For comparison, the full curves show the estimates of the stress–stretch relationship that was obtained from the best emulation method identified in Sections 5.1, 5.2, 5.3, 5.4, the emulation of the outputs method combined with the local GP interpolation method and the Euclidean loss function.

For uncertainty quantification, we numerically estimated the Hessian at the minimum surrogate loss (5). Its inverse represents an approximate lower bound on the variance–covariance matrix in parameter space. (The Hessian is the empirical Fisher information matrix. The lower bound would be exact (Cramer–Rao lower bound) if we could take an expectation with respect to the data distribution. Recall that saying that matrix **A** is a lower bound on matrix **B** means that **B**−**A** is positive semidefinite.) The uncertainty in the estimate can then be obtained by sampling from a multivariate normal distribution, with the covariance set to the inverse of the Hessian, $\text{MVN}\{\hat{\theta },H{\left(\hat{\theta }\right)}^{-1}\}$, and calculating the corresponding confidence intervals.

The results in Fig. 3 show that the emulation method accurately estimates the stretch–stress relationship in the myocyte direction. The agreement between the gold standard and the prediction with our emulation method is nearly perfect, with a deviation that is less than the predicted single‐standard deviation width. For the stretch–stress relationship in the sheet direction, the agreement is also very good, although the deviation exceeds the predicted standard deviation in this case. A possible explanation is that parameter sensitivity in the sheet directions is very low when only using regional circumferential strains and the LV cavity volume to formulate the objective function, as reported in Gao *et al*. (2015); thus the uncertainty of estimating the stiffness in the sheet direction will be higher than that in the myocyte direction. It is expected that higher accuracy will be achieved when radial (transmural) strains are included when inferring the parameters. Although the differences between the stretch–stress curves that were obtained with the simulator and our emulator are minor, there is a substantial difference in the computational costs. For the simulator, i.e. the original procedure that was described in Gao *et al*. (2015) and Gao, Aderhold, Mangion, Luo, Husmeier and Berry (2017), the computational costs are of the order of over a week. The estimation procedure with the emulator proposed, in contrast, could be carried out in less than 15 min (dual Intel Xeon CPU E5‐2699 v3, 2.30 GHz, 36 cores and 128 Gbytes memory), giving us a reduction in the computational complexity by about three orders of magnitude.

Hence, whereas the former procedure is only of interest in a pure research context, the latter procedure gives us estimation times that are acceptable in a clinical decision context. This is an important first step towards bringing mathematical modelling into the clinic and making a real impact in healthcare.

## 6 Discussion

We have developed an emulation framework that can be used to infer the material properties of the left ventricle of a healthy patient in a clinically viable timeframe. We have focused on developing an emulation framework that can be used in future more generalized work and have therefore tested two emulation methods, two interpolation methods and two loss functions; see Section 3. Each combination of these methods has then been evaluated in a simulation study to determine the best method. The best method was found to be the output emulation method, using the local GP as the interpolation method and the Euclidean loss function; see Table 4.

We have then applied the proposed emulation method to cardiac MRI data and demonstrated that it can accurately estimate the stretch–stress relationship along the myocyte and sheet directions of the left ventricle from a healthy volunteer. Our method provides a notable improvement in computational time with a speed‐up of approximately three orders of magnitude. In particular, whereas conventional parameter estimation based on numerical simulations from the mathematical LV model, following for example the approach of Gao *et al*. (2015), leads to computational costs of the order of weeks, the proposed emulation method reduces the computational complexity to the order of a quarter of an hour, while effectively maintaining the same level of accuracy. This is an important step towards a clinical decision support system that can assist a clinical practitioner in realtime.

A limitation of the current approach is the fact that the LV geometry is fixed. This LV geometry varies from patient to patient, and these variations need to be taken into consideration for applications to wider patient populations. We discuss how potentially to address this challenge in the next section.

## 7 Future work

The next step for this work is to design a method that is capable of fast parameter inference for multiple patients on whom we have not directly trained the emulator. For each new patient we would need to replace the single geometry that is used here as an input, with the new patient's data on arrival at the clinic. With no time limits on the inference, we could simply replicate this study with a different input geometry. However, to treat patients in a clinically viable timeframe we must be able to train the emulator for the unobserved patients before they enter the clinic. We can do this by using simulations from multiple LV geometries as our training data. Low dimensional representations of each geometry can then be included as variables in the interpolation method of the emulator and we can learn how these changes affect the output of the biomechanical model. When new patient data then arrive, these low dimensional representations can be calculated and included in the loss function, which must be minimized in the emulation method.

**Figure 4:**
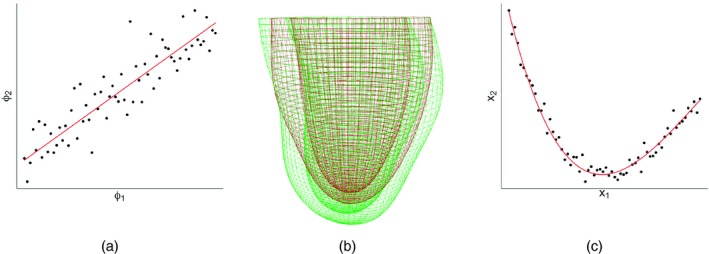
Illustration of dimension reduction for the representation of the left ventricle: (a) illustration of PCA (a set of LV geometries extracted from a set of patients forms a cloud of vectors in a high dimensional vector space (here reduced to 2 for visual representation); PCA provides a set of linear orthogonal subspaces along the directions of maximum variance (here only one, the leading component, is shown)); (b) a variation along the principal component can be mapped back into the high dimensional vector space to show the corresponding changes of the LV geometry (here indicated by different colour shadings); (c) PCA is a linear technique and hence suboptimal if the LV geometries from the patient population are grouped along a non‐linear submanifold

A straightforward approach for achieving this low dimensional representation is principle component analysis (PCA), illustrated in Fig. 4(a), where the high dimensional LV geometries are mapped onto a low dimensional space that captures the maximum variation in the population. A variation along the PCA directions can be mapped back into the high dimensional LV geometry space to illustrate typical organ deformations, as illustrated in Fig. 4(b). However, although fast and easy to implement, the limitation of PCA is its restriction to linear subspaces. If the LV geometries that are extracted from the patient population are grouped along a non‐linear submanifold in the high dimensional LV geometry space, as illustrated in Fig. 4(c), PCA is suboptimal. A variety of non‐linear extensions of and alternatives to PCA have been proposed in the machine learning and computational statistics literature. The most straightforward extension is kernel PCA (Scholkopf *et al*., 1998), which conceptually maps the data non‐linearly into a high dimensional vector space and makes use of Mercer's theorem, whereby the scalar product in this high dimensional space is equivalent to a kernel in the original data space and therefore never has to be computed explicitly. Alternative non‐linear dimension reduction methods to be explored are generative topographic maps (Bishop *et al*., 1998), self‐organizing maps (Kohonen, 1982) and variational autoencoding neural networks (Kingma and Welling, 2014).

## 8 Software

The software developed for and used in our study can be downloaded from https://github.com/vinnydavies/left-ventricle-jrss-c.

## Supporting information

‘Fast parameter inference in a biomechanical model of the left ventricle using statistical emulation’.
