# Supplementary material for: Fast parameter inference in a biomechanical model of the left ventricle by using statistical emulation
Source: J R Stat Soc Ser C Appl Stat. 2019 Sep 20;68(5):1555–76. doi: 10.1111/rssc.12374 (PMC6856984; doi:10.1111/rssc.12374)
Supplement: Supplementary file 1 — ‘Fast parameter inference in a biomechanical model of the left ventricle using statistical emulation’. [file RSSC-68-1555-s001.pdf]

Fast Parameter Inference in a Biomechanical Model of the Left Ventricle using Statistical Emulation (Supplementary Material)

Vinny Davies[*,1,2], Umberto Noè[*,3], Alan Lazarus[2], Hao Gao[2], Benn Macdonald[2], Colin Berry[4,5], Xiaoyu Luo[2], Dirk Husmeier[2]

[*]Contributed equally.
[1]School of Computing Science, University of Glasgow, Glasgow, UK.
[2]School of Mathematics and Statistics, University of Glasgow, Glasgow, UK.
[3]German Centre for Neurodegenerative Diseases (DZNE), Bonn, Germany.
[4]BHF Glasgow Cardiovascular Research Centre, University of Glasgow, Glasgow, UK.
[5]West of Scotland Heart and Lung Centre, Golden Jubilee National Hospital, Clydebank, UK.

This online supplementary details provides additional details that were omitted from the main paper for brevity. Section 1 gives details of how the parameter inference is evalutated in real data. Section 2 gives details of the parameter reduction used to reducer the complexity of the differential equation that forms part of the biomechanical model. Section 3 provides additional figures that were not included in the main paper.

1 Evaluating Inferred Myocardial Properties

As a result of the nonlinear hyperelastic response of the myocardium and non-uniqueness of the material parameters estimated from experimental data, we use the stress–strain relationship along a specific material axis to represent myocardial stiffness. The change of the shape is described by the deformation gradient $\mathbf{F}$. The deformed myofibre direction at the currenct configuration is $\mathbf{F}\,\mathbf{f}_0$, in which $\mathbf{f}_0$ is the myofibre direction at the reference state, and the myofibre stretch is $|\mathbf{f}|$. If the corresponding stress tensor is $\boldsymbol{\sigma}$, then the stress along the myofibre direction is $\mathbf{f}_0 \cdot (\boldsymbol{\sigma}\,\mathbf{f}_0)$. In this study, we plot the stress as a function of stretch along the myofibre and the sheet directions to represent the LV material properties. An example is shown in Figure 1a. We see that the relationship between stretch and stress is nonlinear. If a stretch-stress curve is above another one, then the corresponding myocardial stiffness is higher than the one associate with the curve below. For example, in Figure 1, myocardial stiffness associated with the solid blue curve is higher than the one associated with the black dashed curve.

2 Reduced Four-Dimensional Parameter Space

In our previous study (Gao et al., 2017), we inversely estimated the 8 passive material parameters for 27 healthy volunteers. Here we project all estimated values to a lower-dimensional representation, $\theta_1, \theta_2, \theta_3$, and θ_4, by minimizing the differences between the full set and the

dimension-reduced parameters,

$$
\begin{cases}
\theta_1^{\text{full}} = \arg\,\min_{\theta_1} \left\{ || \frac{a^{\text{full}} - a_0 \theta_1}{a_0} ||^2 + || \frac{b^{\text{full}} - b_0 \theta_1}{b_0} ||^2 \right\} \\
\theta_2^{\text{full}} = \arg\,\min_{\theta_2} \left\{ \frac{|| a_{\text{f}}^{\text{full}} - a_{\text{f}0}\, \theta_2}{a_{\text{f}0}} ||^2 + \frac{|| a_{\text{s}}^{\text{full}} - a_{\text{s}0}\, \theta_2}{a_{\text{s}0}} ||^2 \right\} \\
\theta_3^{\text{full}} = \arg\,\min_{\theta_3} \left\{ \frac{|| b_{\text{f}}^{\text{full}} - b_{\text{f}0}\, \theta_3}{b_{\text{f}0}} ||^2 + \frac{|| b_{\text{s}}^{\text{full}} - b_{\text{s}0}\, \theta_3}{b_{\text{s}0}} ||^2 \right\} \\
\theta_4^{\text{full}} = \arg\,\min_{\theta_4} \left\{ || \frac{a_{\text{fs}}^{\text{full}} - a_{\text{fs}0} \theta_4}{a_{\text{fs}0}} ||^2 + || \frac{b_{\text{fs}}^{\text{full}} - b_{\text{fs}0} \theta_4}{b_{\text{fs}0}} ||^2 \right\}
\end{cases}
\tag{1}
$$

in which $[\bullet]^{\text{full}}$ denotes the estimated parameters from Gao et al. (2017) based on the multi-step optimization approach desribed in Gao et al. (2015). We then compared the stretch-stress relationship under uni-axial stretch along the myofibre direction for a healthy volunteer, as shown in Figure 1. The stretch-stress relationship using the projected parameter space is nearly identical to that obtained with the estimated parameters from Gao et al. (2017), and the mean stress difference, calculated by the area-under-the-curve with stretches from 1.0 to 1.2, is 2.1% for all 27 subjects. This suggests that the 8 parameters can be reduced to a 4-dimensional representation with negligible information loss, (1), and that the reduced parameter representation can still describe the myofibre stretch-stress relationship accurately.

3 Supplementary Figures

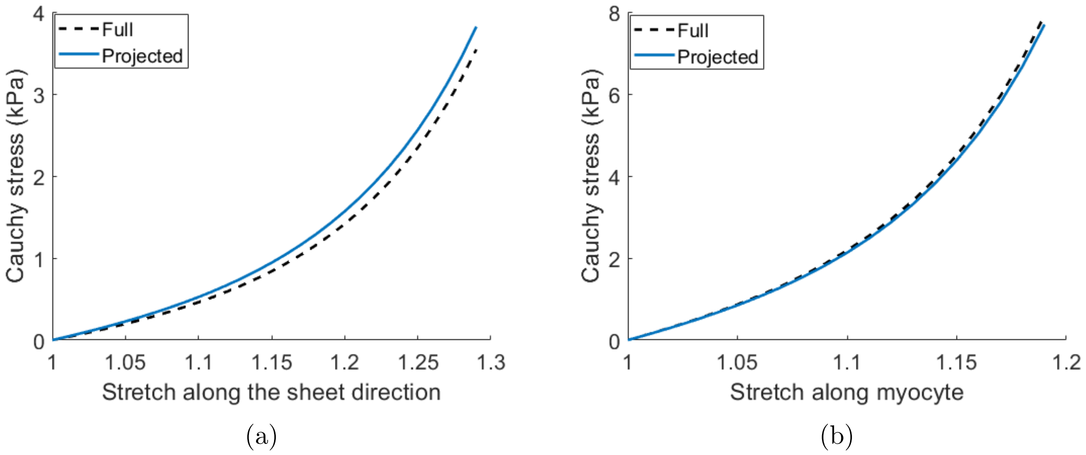

Figure 1: Plots of the Cauchy stress against the stretch along (a) the sheet direction and (b) the myocytes. The black dashed lines show the resulting plots when the full 8-dimensional parameter set is used. The solid blue lines show the resulting plots for a reduced 4-dimensional parameter space.

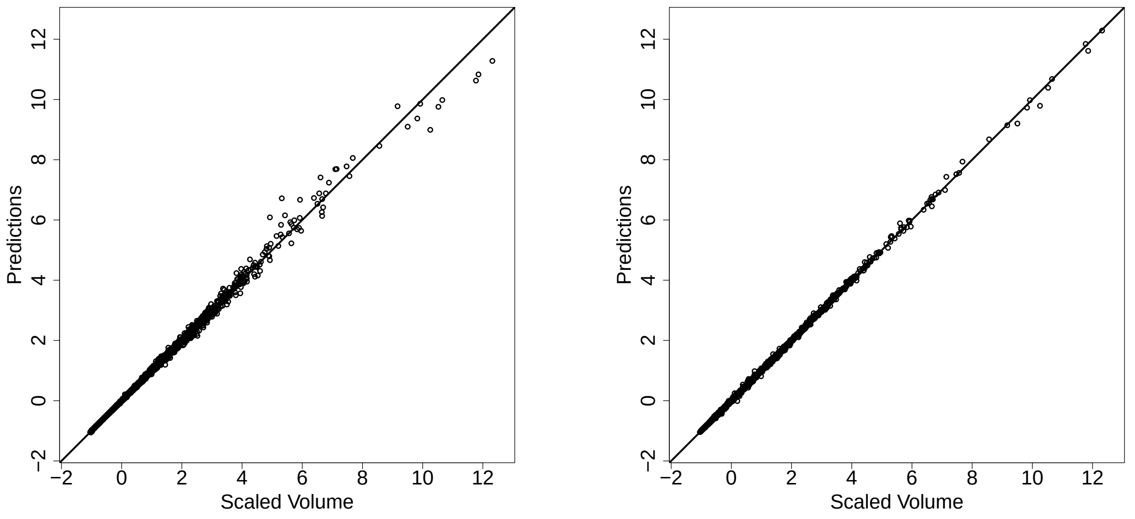

(a) GP model without inverse term

(b) GP model with inverse term

Figure 2: Predicted values of the in-sample scaled LV volume data using the low rank GP method, Section 3.4.2 in the main paper, versus the true values. Results are shown for (a) a GP regression model with an interactive term between the model parameters, $\boldsymbol{\theta}$, and (b) a GP regression model with two terms, an interactive term between the model parameters, $\boldsymbol{\theta}$, and an interactive term between the inverse of these model parameters. Alternative plots for out-of-sample data are given in Figure 3 below.

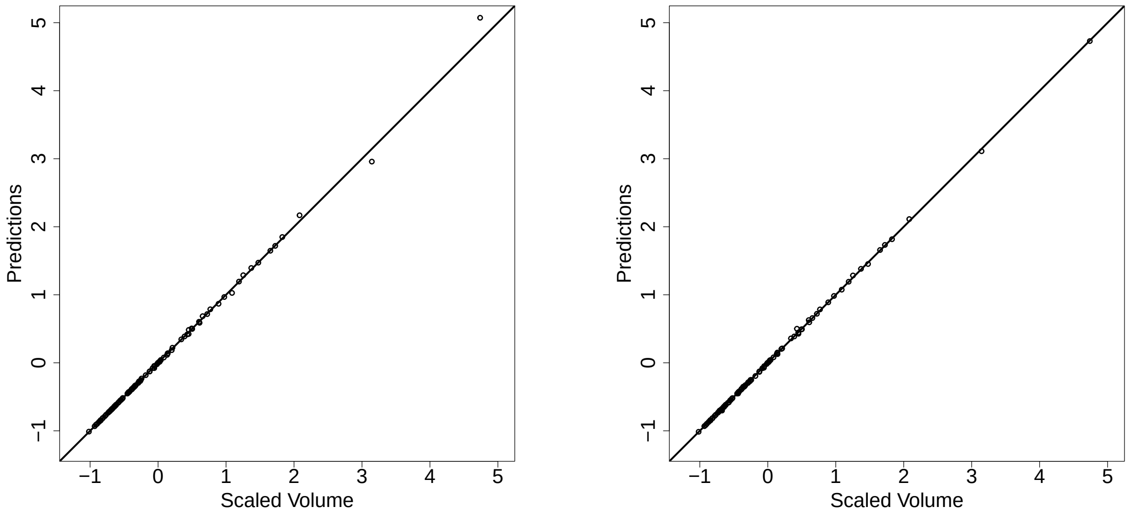

(a) GP model without inverse term

(b) GP model with inverse term

Figure 3: Predicted values of the out-of-sample scaled LV volume data using the low rank GP method, Section 3.4.2 in the main paper, versus the true values. Results are shown for (a) a GP regression model with an interactive term between the model parameters, $\boldsymbol{\theta}$ and (b) a GP regression model with two terms, an interactive term between the model parameters, $\boldsymbol{\theta}$, and an interactive term between the inverse of these model parameters. Alternative plots for in-sample data are given in Figure 2 above.

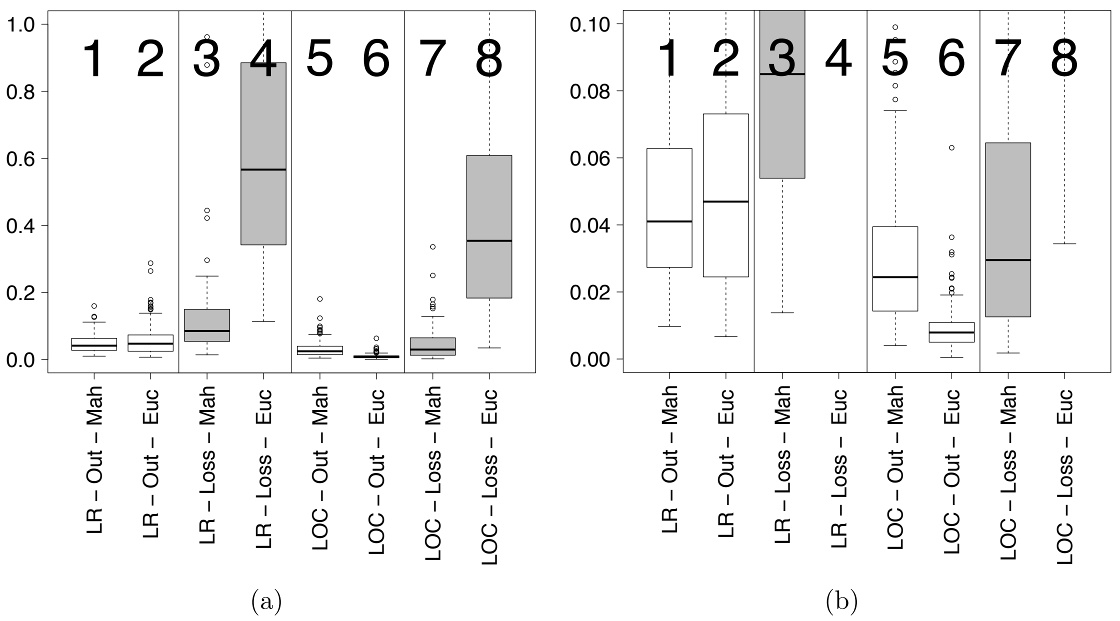

Figure 4: Boxplots giving the mean absolute error (in parameter-space) in the prediction of all the model parameters. Panel (a) gives boxplots of the mean absolute error for all the 8 methods, panel (b) gives the same boxplots but with a reduced scale on the y-axis. The methods from left to right on each plot are as follows: low rank GP (LR) output emulation (Out) with Mahalanobis loss function (Mah) and Euclidean loss function (Euc), LR-GP loss emulation (Loss) with Mahalanobis loss function and Euclidean loss function, local GP (LOC) output emulation with Mahalanobis loss function and Euclidean loss function, and LOC loss emulation with Mahalanobis loss function and Euclidean loss function.

References

Gao, H., A. Aderhold, K. Mangion, X. Luo, D. Husmeier, and C. Berry (2017). Changes and classification in myocardial contractile function in the left ventricle following acute myocardial infarction. *Journal of The Royal Society Interface 14*(132), 20170203.

Gao, H., W. Li, L. Cai, C. Berry, and X. Luo (2015). Parameter estimation in a Holzapfel–Ogden law for healthy myocardium. *Journal of engineering mathematics 95*(1), 231–248.
